# Investigating cortico-striatal beta oscillations in Parkinson’s disease cognitive decline

**DOI:** 10.1093/brain/awad273

**Published:** 2023-08-14

**Authors:** Mansoureh Fahimi Hnazaee, Vladimir Litvak

**Affiliations:** Wellcome Centre for Human Neuroimaging, UCL Queen Square Institute of Neurology, London, UK; Wellcome Centre for Human Neuroimaging, UCL Queen Square Institute of Neurology, London, UK

## Abstract

This scientific commentary refers to ‘Corticostriatal beta oscillation changes associated with cognitive function in Parkinson’s disease’ by Paulo *et al.* (https://doi.org/10.1093/brain/awad206).

**This scientific commentary refers to ‘Corticostriatal beta oscillation changes associated with cognitive function in Parkinson’s disease’ by Paulo *et al.* (https://doi.org/10.1093/brain/awad206).**

The basal ganglia, seated at the base of the forebrain, are a set of subcortical structures dating back phylogenetically at least 500 million years, and therefore sharing many of their organizational principles with equivalent structures in the brains of primates, rodents and even certain classes of lower vertebrates.^1^ The basal ganglia maintain reciprocal connections with distinct areas in the brainstem, cerebellum, thalamus and cortex with the likely primary function of executing automatic behaviours across the motor and cognitive domains. Our existing models of basal ganglia circuitry emerged from animal studies, with later research focusing on the translation of these models into clinical treatment. Invasive human neurophysiological recordings during or after functional neurosurgery have provided insights into the role of cortico-basal ganglia circuits in neurological diseases, including Parkinson’s disease and Alzheimer’s disease.^2^ Studies of neural oscillations and coupling have uncovered mechanisms of behaviour and pathology, with studies in humans having established a key role for low beta oscillations in Parkinson’s disease motor impairment.^3^

However, less is known about cognitive impairments caused by these diseases, particularly in executive functions such as attention, long-term memory, and working memory. These impairments pose at least as significant a problem as motor-related symptoms but have not been studied as extensively. Neuromodulation techniques such as deep brain stimulation (DBS) can help reduce these impairments, by applying electrical current to specific brain network components involved in cognitive functions. Current research seeks to identify novel targets related to memory,^4^ focusing on the segregated basal ganglia thalamocortical circuit involving the caudate and frontal cortex. Dysfunction in this circuit may contribute to cognitive slowing in Alzheimer’s disease, though the parallels with cognitive impairment in Parkinson’s disease remain unclear (Fig. 1).

**Figure 1:**
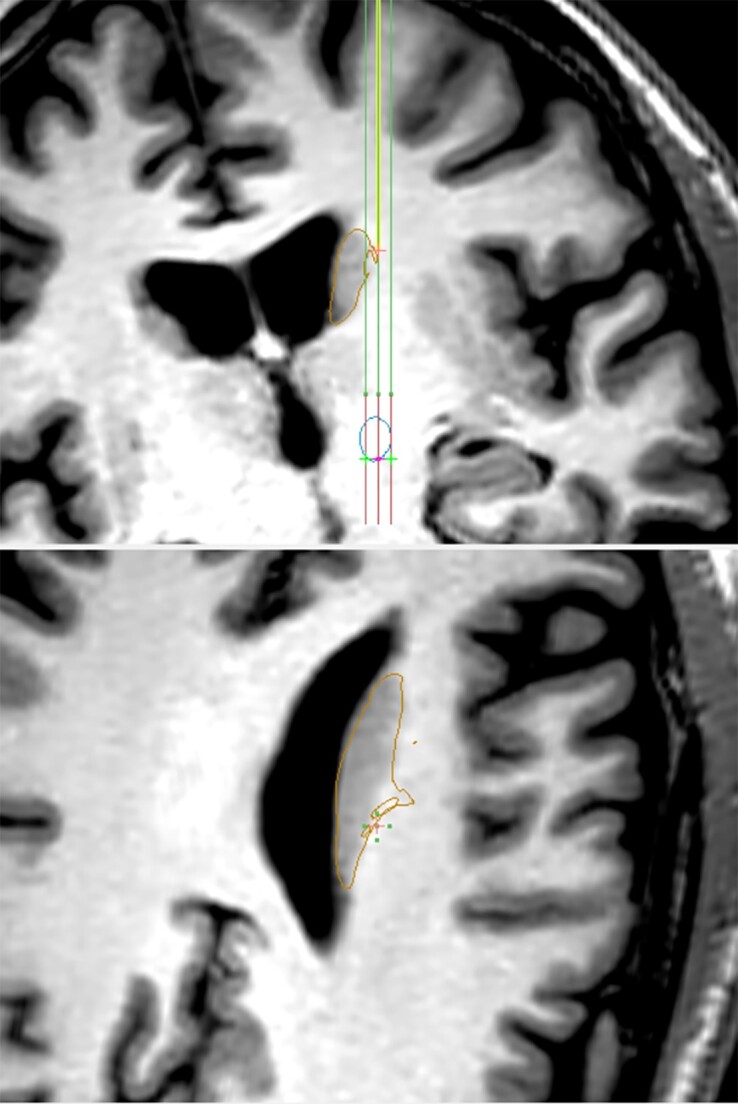
**DBS trajectory with depth of recording sampled from the caudate, during the experiments by Paulo *et al*.^5^** Caudate is outlined in brown; STN—the clinical target—is outlined in blue; BenGun trajectory/tracks are shown in green.

In this issue of *Brain*, Paulo and co-workers^5^ use DBS to investigate the mechanisms underlying cognitive impairment in Parkinson’s disease. Their innovative approach involved recording directly from the caudate and the dorsolateral prefrontal cortex (DLPFC) of participants during a working memory *n*-back task. While the caudate and DLPFC were not the targets of the surgery, they were located along the planned electrode trajectory to the targets—the subthalamic nucleus and internal pallidum—which meant that the experiment could be performed under local anaesthesia, during insertion of the DBS electrode. The practicality and feasibility of such recordings must be assessed on an individual basis by the medical team, given that traversing the caudate is generally avoided. However, given the experiment’s brief duration of ∼16 min including the resting state, it is plausible that such opportunities could be exploited more frequently. This could allow for unique neurophysiological insights, typically inaccessible in human studies, that could yield pivotal advances in our understanding of pathological neural circuits.^6^

In the current study, macro-electrode recordings across various sites in both the caudate and DLPFC revealed a decrease in beta power during encoding relative to the resting state. This observation in and of itself is a significant contribution to our understanding of working memory mechanisms. Previous reports have noted beta modulations during working memory activities using EEG and MEG, though it remains unclear whether these modulations differ functionally from those in the alpha band, or if they simply span both bands.^7^ Additionally, EEG and MEG have less anatomical precision compared to local field potential recordings. In particular, the ability of these non-invasive techniques to capture signals from subcortical structures such as the basal ganglia is subject to discussion. The discovery of beta modulations specific to the caudate, with caudate beta power significantly reduced during encoding in correct trials but not incorrect ones, could also inform and validate future source reconstruction studies of working memory using EEG and MEG.

Comparing recordings between cognitively healthy individuals and those with mild cognitive impairment revealed a smaller decrease in beta power in the caudate and DLPFC during encoding in the group with mild cognitive impairment. The role of pathological oscillations in cognitive impairment in Parkinson’s disease has to date received less attention than that of pathological oscillations in motor impairment. Both the caudate and DLPFC displayed decreased beta oscillations during memory encoding, and increased oscillations during feedback. The smaller reduction in beta observed during memory encoding (but not feedback) in individuals with cognitive impairment suggests potential dysfunction in the cognitive cortico-striatal circuit. Further exploratory analyses revealed similar between-group differences during encoding in the alpha band in both regions and in theta in the DLPFC. During the feedback stage, the only difference between individuals with and without cognitive impairment was in the alpha band in the DLPFC, hinting at a distinct dysfunction in reward processing. This aligns with studies highlighting the involvement of medial and lateral prefrontal cortex in conveying motivational incentives.^8^

The study was constrained by the fact that only patients with mild cognitive impairment could participate, as severe cognitive impairment disqualifies individuals from DBS surgery. This restriction might explain the lack of any significant difference in caudate volume between the two groups, even though previous research has revealed lower caudate volumes in individuals with mild cognitive impairment and Alzheimer’s disease.^9^ Similarly, the current study did not find a significant correlation between caudate volume and beta power in either the caudate or DLPFC. It is also worth noting that no significant difference in task performance between the groups was reported by the authors, suggesting the task might not have been challenging enough to highlight the full spectrum of neurophysiological differences between cognitively healthy patients with Parkinson’s disease and those with mild cognitive impairment.

Owing to the experimental set-up, simultaneous recording from both the caudate and DLPFC within the same hemisphere was not possible, as the electrode trajectory passed through one region or the other. This constraint prevented the study from determining whether there was functional coupling or directional information transfer between these regions. The authors chose not to examine interhemispheric coupling, likely due to the heterogeneous nature of the recordings. Future studies could augment this design by incorporating intraoperative recording from the caudate along the electrode trajectory and combining it with non-invasive measurements of the DLPFC, which can be obtained, albeit less precisely, through surface EEG. Identifying the network characteristics of cognitive functions will aid in the development of more finely targeted DBS strategies. Merging invasive with non-invasive techniques is crucial for a comprehensive view of cortical brain regions and their basal ganglia connections.^10^

As well as identifying a potential novel DBS target for cognitive impairment in Parkinson’s disease, this work has uncovered caudate and DLPFC biomarkers for use with closed-loop strategies. As the technology for DBS continues to improve, with directional leads and closed-loop stimulation increasing treatment effectiveness, and the development of better algorithms allowing for greater exploration of the massive parameter space to optimize programming, the importance of identifying novel targets to allow DBS to be applied to additional disease indications is clear.
